# Emergent initial presentation of congenital malrotation with midgut volvulus in 68-year-old

**DOI:** 10.1093/jscr/rjad078

**Published:** 2023-03-07

**Authors:** Maria E Tecos, Margarita Pipinos, Brett H Waibel

**Affiliations:** Department of General Surgery, University Of Nebraska Medical Center, Omaha, NA 68198, USA; Department of General Surgery, University Of Nebraska Medical Center, Omaha, NA 68198, USA; Department of General Surgery, University Of Nebraska Medical Center, Omaha, NA 68198, USA

**Keywords:** malrotation, midgut volvulus, congenital anomaly, geriatric surgery, pediatric surgery, emergency general surgery

## Abstract

Congenital malrotation is a pathology nearly exclusive to the infant population. In the rare instance when it is diagnosed in an adult, it is typically associated with a longstanding history of gastrointestinal symptoms. Unfortunately, this unique presentation in an unexpected population has the potential to be confounding, leading to delayed or mismanaged care. Here, we describe an intriguing case of congenital malrotation complicated by midgut volvulus in a 68-year-old woman. Even more curious, the patient did not have a medical history plagued by abdominal complaints. Careful, comprehensive evaluation yielded appropriate surgical management via Ladd’s procedure and right hemicolectomy in this complex patient.

## INTRODUCTION

The failure of the small intestine to rotate counterclockwise about the axis of the superior mesenteric artery is a developmental anomaly nearly exclusively diagnosed in early infancy or childhood (>99.5% of cases). Of the exceedingly rare instances of adult presentation, the pathology progresses to midgut volvulus in only 15% of occurrences. Midgut volvulus can be considered a surgical emergency due to the potential of massive bowel ischemia, with standard operative management being Ladd’s procedure [1]. Here, we detail the diagnosis and operative management of a 68-year-old female with newly diagnosed congenital malrotation with midgut volvulus, with the unique presentation of no prior chronic abdominal symptoms.

### CASE REPORT

The patient was a 68-year-old female with a past medical history including atrial fibrillation. She had no prior abdominal surgeries. She was referred after she developed vague abdominal pain in the morning, which worsened within an hour of eating lunch in the afternoon. The pain was present diffusely about her abdomen and radiated to her back. It was associated with a single episode of nausea and vomiting, which did not improve her pain. Her last bowel movement had been that morning and was without blood. She had no longstanding history of prior abdominal symptoms, bilious emesis or any prior episode of pain similar to her current presentation.

**Figure 1:**
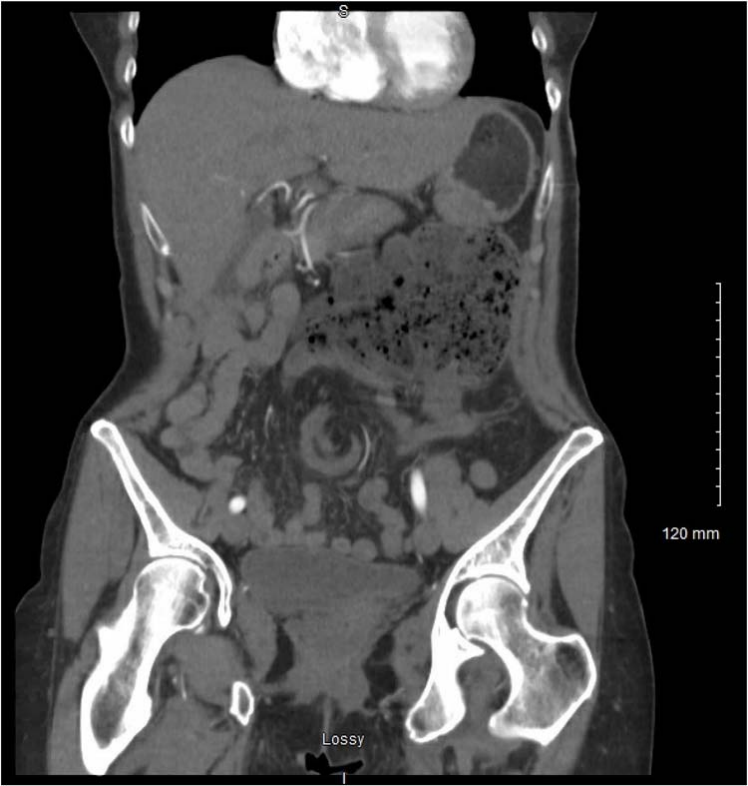
Coronal view of the patient’s CT abdomen and pelvis showing a central mesenteric swirl and adjacent fecalised bowel concerning for volvulus.

Her persistent pain prompted her to seek further care via the emergency department where computerised tomography (CT) of the abdomen and pelvis revealed concern for congenital malrotation with closed-loop bowel obstruction secondary to intestinal volvulus of the cecum and ascending colon (Fig. 1). At this time, vital signs were found to be within normal limits. White blood cell count was found to be 7.9 and lactate was slightly elevated at 2.6. Her abdomen was tender in the left upper quadrant, with a palpable fullness underlying the area of maximum tenderness. She did not have peritoneal signs on abdominal exam. Based on these findings, the decision was made to proceed to the operating room for exploratory laparotomy.

**Figure 2:**
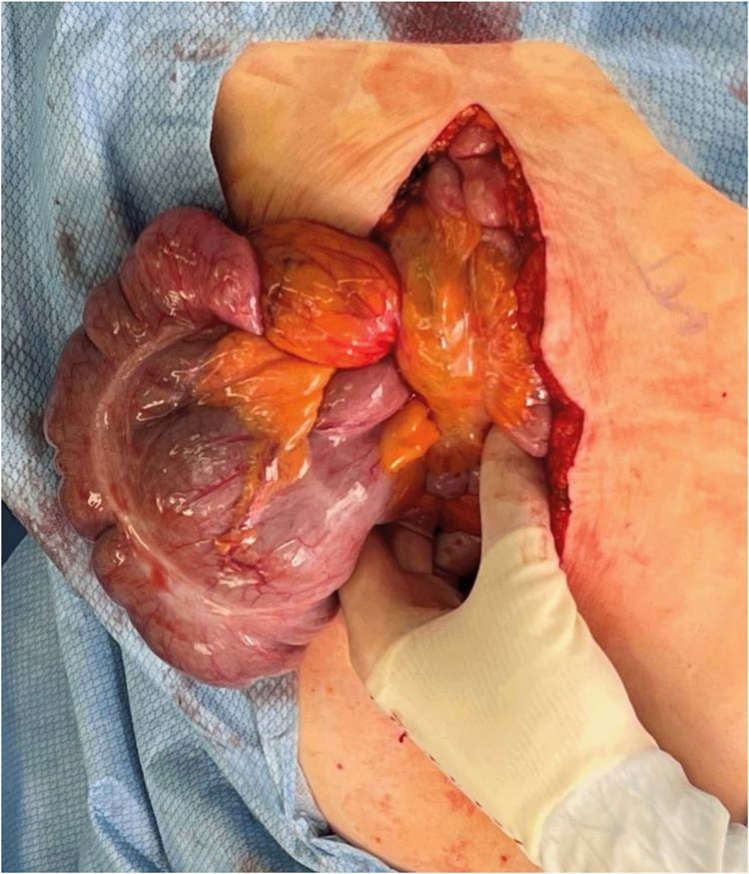
Evisceration of the midgut volvulus upon entry into the abdomen.

**Figure 3:**
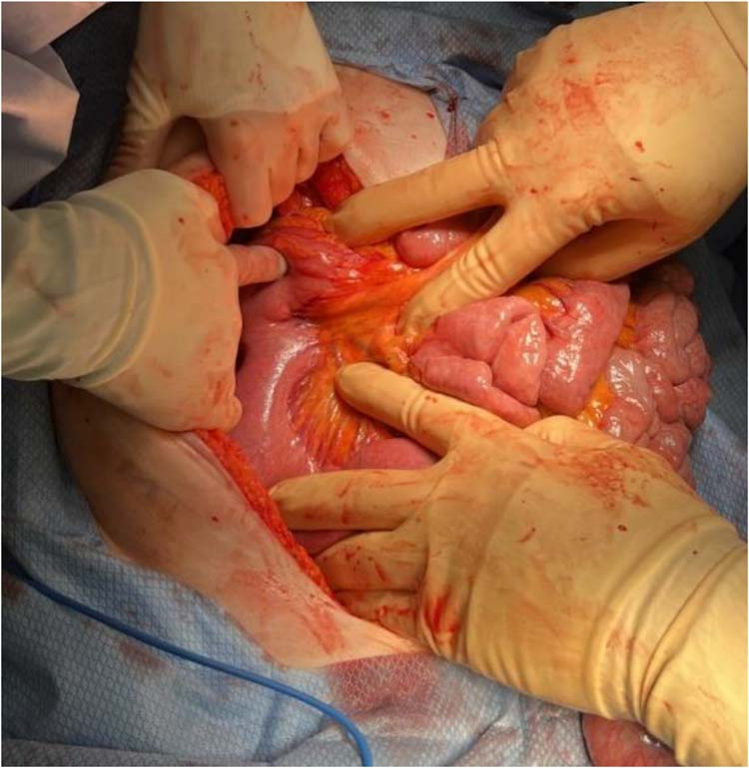
Identification of Ladd’s bands and lack of retroperitoneal course of the duodenum after detorsion of the midgut volvulus.

A midline laparotomy was made. A midgut volvulus was encountered immediately upon entering the abdomen and was subsequently reduced from the left upper quadrant. The cecal and ascending colon volvulus had a 540 degree rotation with viable tissues on reduction; however, the ascending colon had a narrow, non-adherent mesentery allowing a point of volvulus while also folding over a Ladd’s band attached to the right lower quadrant of the abdomen. The bowel was then run, and it was confirmed that there was no present ligament of Treitz and that the patient did indeed have congenital malrotation (Fig. 2). There was no retroperitoneal component of duodenum, and it was completely without fixation to other structures. Ladd’s bands were encountered and lysed (Fig. 3).

The mesentery of the small bowel did appear to be adequately broad. As this was an atypical presentation of malrotation with midgut volvulus, and the cecum did appear abnormally dilated, we did elect to perform a right hemicolectomy during this operation to remove an additional nidus around which re-volvulus might occur. A proximal resection point in the terminal ileum approximately 20 cm from the ileocecal valve was anastomosed with a segment of colon just past the hepatic flexure where the colon caliber had returned to normal; the intervening bowel was resected and the mesenteric defect was closed. The bowel was then run again and was without any necrotic or ischemic segments. It was confirmed that all present Ladd’s bands had been lysed. The small intestine was placed in the right hemiabdomen and the remaining colon was placed in the left hemiabdomen. The patient tolerated the procedure well, without any complication.

Post-operatively, her presenting abdominal pain and nausea were resolved. The patient’s lactate reduced to 1.7 and she remained vitally stable. Her pain was well controlled, she was able to ambulate about the floor, and she tolerated a progressively advanced diet without nausea or vomiting. She had spontaneous return of bowel function and was discharged to home in good condition on post-operative day 5.

## DISCUSSION

Congenital malrotation diagnosed in the elderly adult population is exceedingly uncommon [1]. Further, its diagnosis in a patient without any history of prior abdominal complaints is quite unique. In non-infant diagnosis of congenital malrotation, the presentation typically consists of chronic symptoms often characterised by colicky abdominal pain, bilious or nonbilious emesis, weight loss and food intolerance [2, 3]. The average age of adult diagnosis ranges from late 30s to early 40s, with scant reports of newly diagnosed cases in patients as old as 80s [2, 4–14]. Remarkably, rates of surgery for adult-diagnosed malrotation can be less than 15%, with few adult patients ultimately undergoing the recommended Ladd’s procedure for this pathology [15]. Delayed or missed diagnosis in the unexpected adult population can lead to increased morbidity and mortality, as well as inadequate or inappropriate cares [14, 15].

The intrigue of this case lies not only in the rare presentation of pediatric pathology in a geriatric patient, but is further imbued the combination of surgical principles from both population that was employed to optimally treat this patient. In the pediatric population, preservation of viable bowel is paramount. However, in this patient, in her seventh decade of life, preserving abnormally dilated bowel with a malformed mesentery presented a risk for re-volvulus. For this reason, the surgical planning evolved from a standard Ladd’s procedure to include a right hemicolectomy. This thoughtful surgical consideration was of the utmost importance, considering the lack of appropriate operative management for congenital malrotation in the adult population [15].

For this patient, comprehensive analysis of the constellation of her symptoms in the setting of her distinctive radiological findings led to the swift and accurate identification of an unlikely pathology. Careful consideration of her unique circumstance as well as the surgical approaches best suited to her anatomy and potential complications allowed for optimal management of her complex ailment.
